# Supplementary material for: A weighted constraint satisfaction approach to human goal-directed decision making
Source: PLoS Comput Biol. 2022 Jun 16;18(6):e1009553. doi: 10.1371/journal.pcbi.1009553 (PMC9255770; doi:10.1371/journal.pcbi.1009553)
Supplement: S1 Text — Use of additional fitting runs to avoid local minima. (PDF) [file pcbi.1009553.s001.pdf]

## Supplementary Methods

**Using additional fitting runs to avoid local minima.** For the winning group model in Experiment 1, we observed that some runs under each fold converged to what appeared to be a local minimum value of the summed negative log-likelihood (sNLL) of the fold training data with near-zero starting point variability ($sz$), as in practice a large $sz$ always resulted in better data likelihood compared to a near-zero $sz$. For five of the cross-validation folds, all ten runs with different initial parameter values converged to this local minimum. In these cases, we ran 50 more runs each using different random samples of initial parameter values to find a set of final parameter values better than those at this local minimum. This was successful; the run with the smallest training sNLL across all 60 runs in all five cases had a larger $sz$ value within the same range of values arising from the other 195 folds. The parameter estimates of the new best-fitting runs for these five folds are reported in the paper. For the same reason, when fitting the model to data from Experiment 1's higher accuracy group, we used an initial set of 50 independent runs (instead of 10) for each fold in order to robustly find large $sz$'s. We conducted additional rounds of model fitting (50 runs at a time) for the winning model in a few folds that converged to local minima with near-zero $sz$'s after the initial runs (eight folds for Experiment 1's higher accuracy group, three folds for Experiment 1's lower accuracy group). For Experiment 2, model selection was restricted to only an initial set of 10 runs for each fold with no additional fitting runs performed.
