# Supplementary material for: A weighted constraint satisfaction approach to human goal-directed decision making
Source: PLoS Comput Biol. 2022 Jun 16;18(6):e1009553. doi: 10.1371/journal.pcbi.1009553 (PMC9255770; doi:10.1371/journal.pcbi.1009553)
Supplement: S1 Fig — Visualization and notation as in Fig 3. The individual median zscore response times were projected to the raw time scale in seconds using the subgroup-average of mean response time and standard deviation. (PDF) [file pcbi.1009553.s002.pdf]

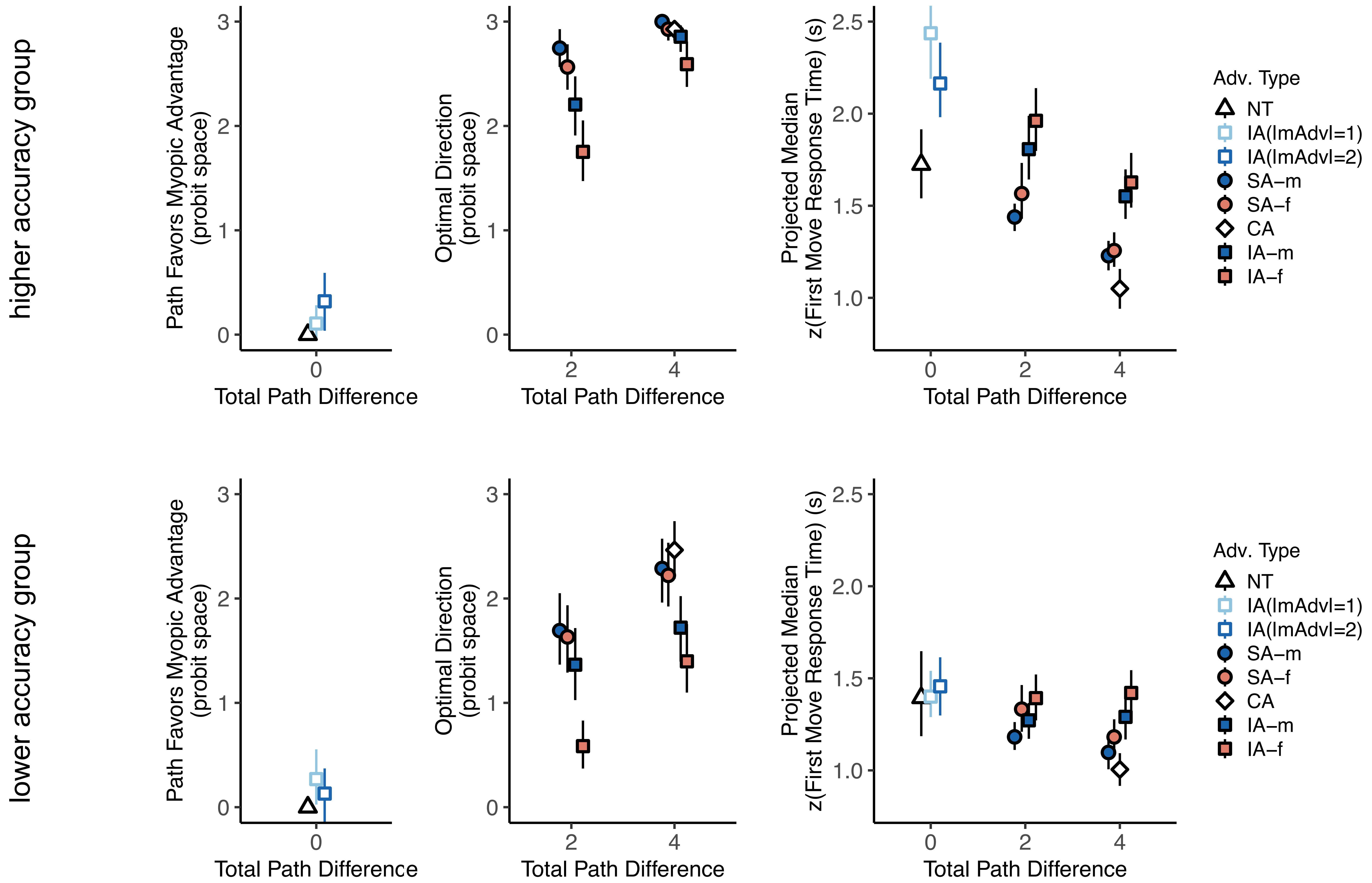

higher accuracy group
lower accuracy group
Path Favors Myopic Advantage (probit space)
Optimal Direction (probit space)
Projected Median z(First Move Response Time) (s)
Total Path Difference
Adv. Type
NT
IA(ImAdvl=1)
IA(ImAdvl=2)
SA−m
SA−f
CA
IA−m
IA−f
