# Supplementary figures and images for: A weighted constraint satisfaction approach to human goal-directed decision making
Source: PLoS Comput Biol. 2022 Jun 16;18(6):e1009553. doi: 10.1371/journal.pcbi.1009553 (PMC9255770; doi:10.1371/journal.pcbi.1009553)

higher accuracy group

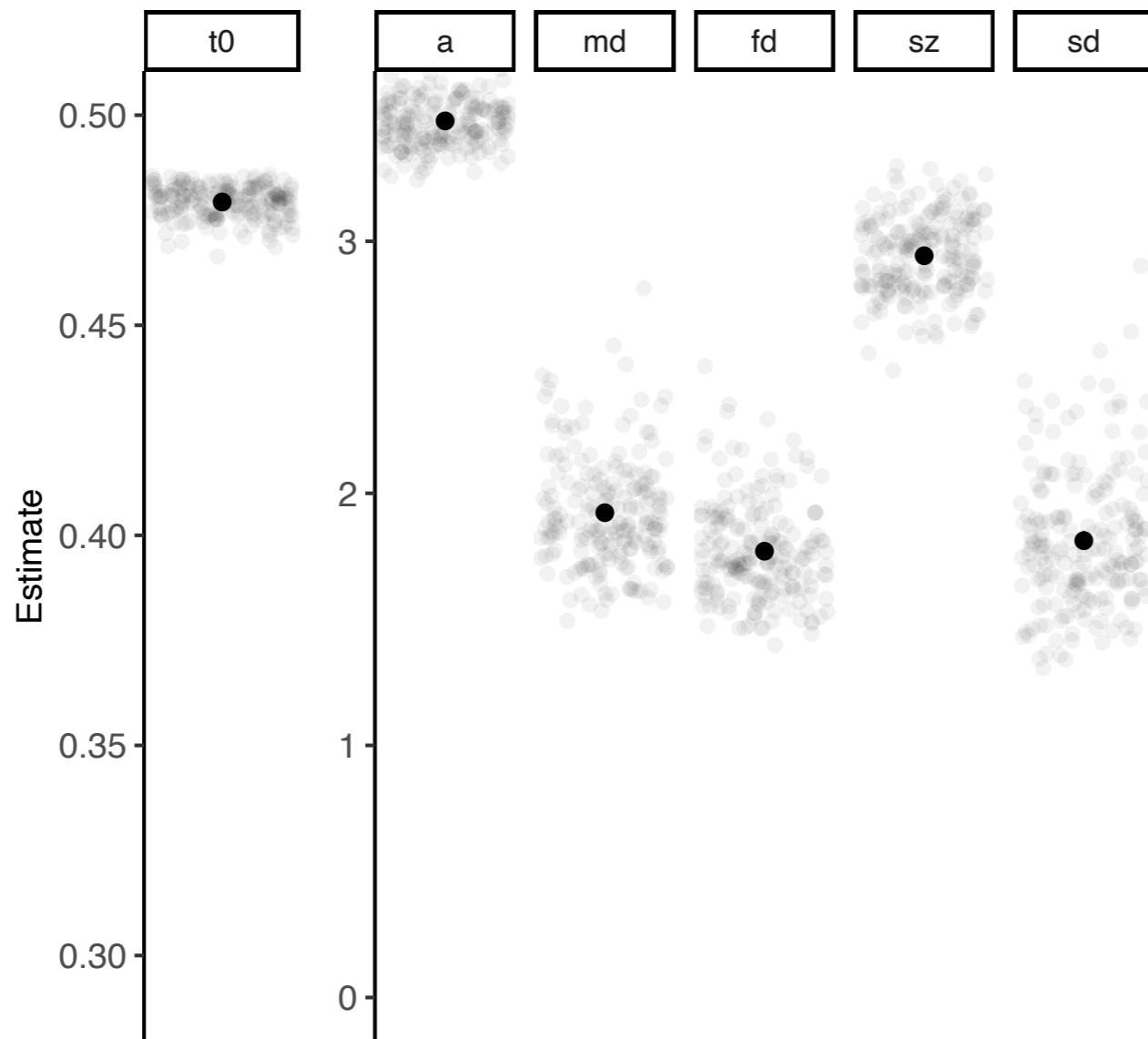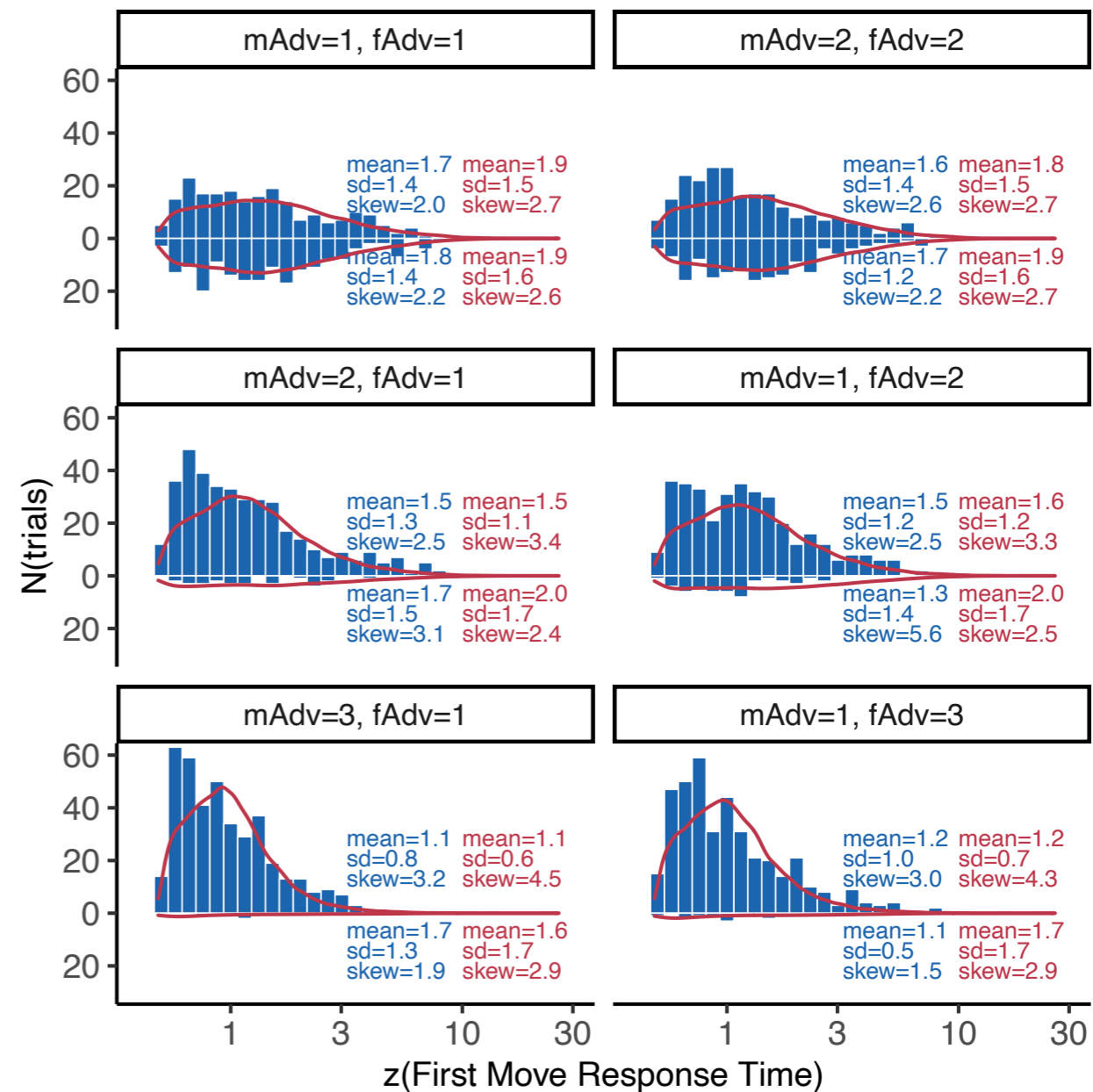

lower accuracy group

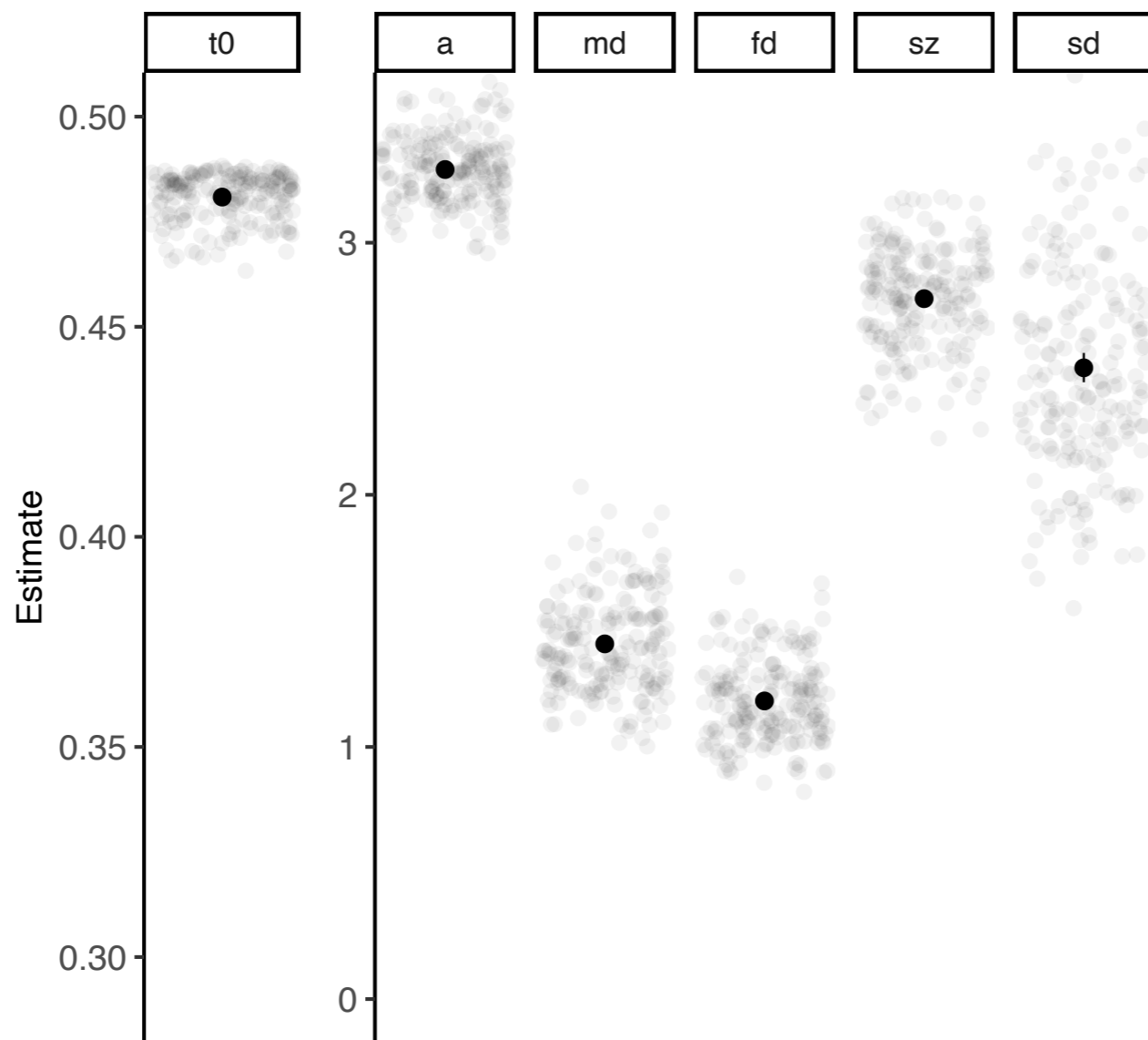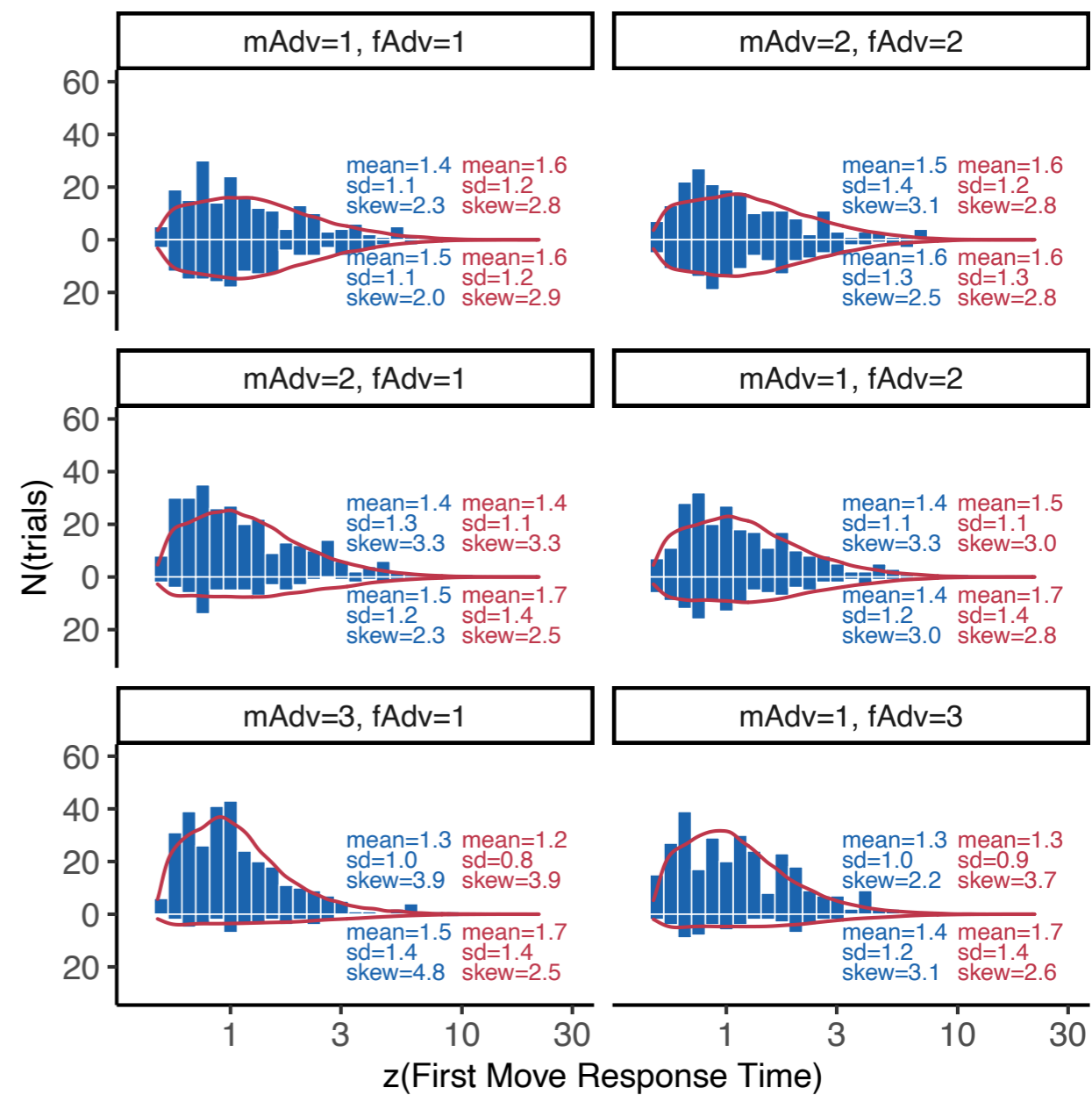

Supplement: S2 Fig — Visualization and notation as in Fig 4B and 4C. (PDF) [file pcbi.1009553.s003.pdf]

higher accuracy group

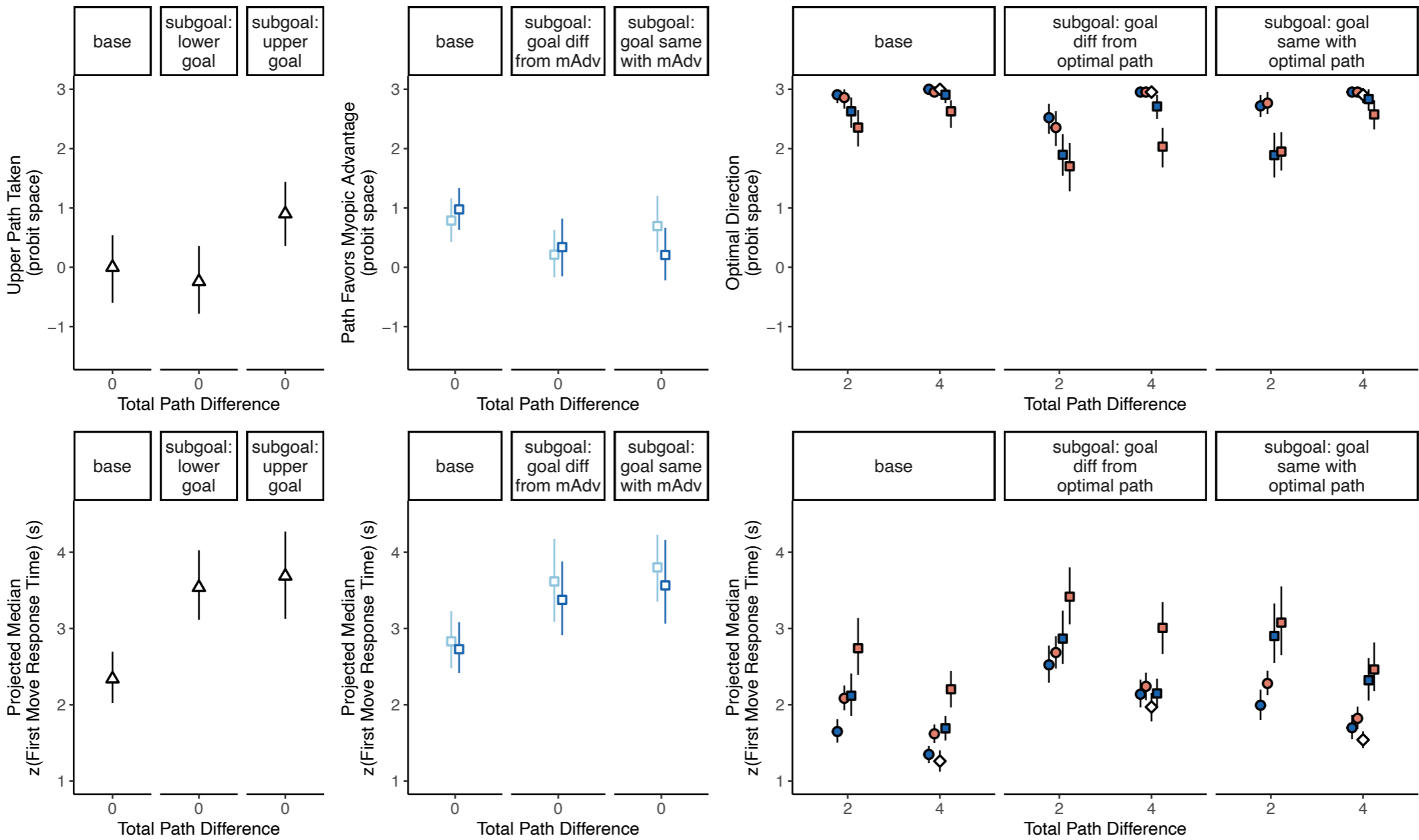

lower accuracy group

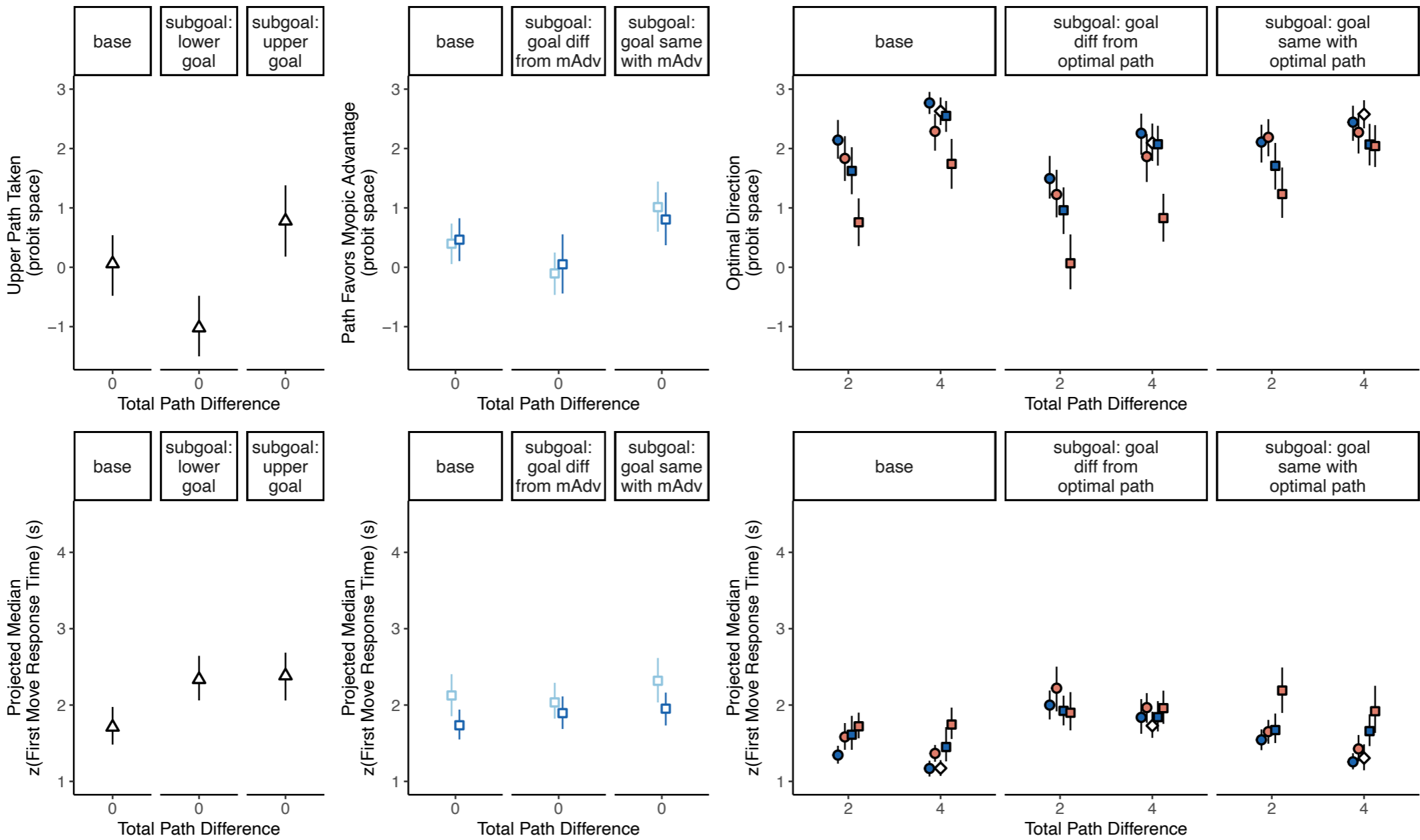

Supplement: S3 Fig — Visualization as in Fig 5. The individual median zscore response times were projected to the raw time scale in seconds using the subgroup-average of mean response time and standard deviation. (PDF) [file pcbi.1009553.s004.pdf]

A

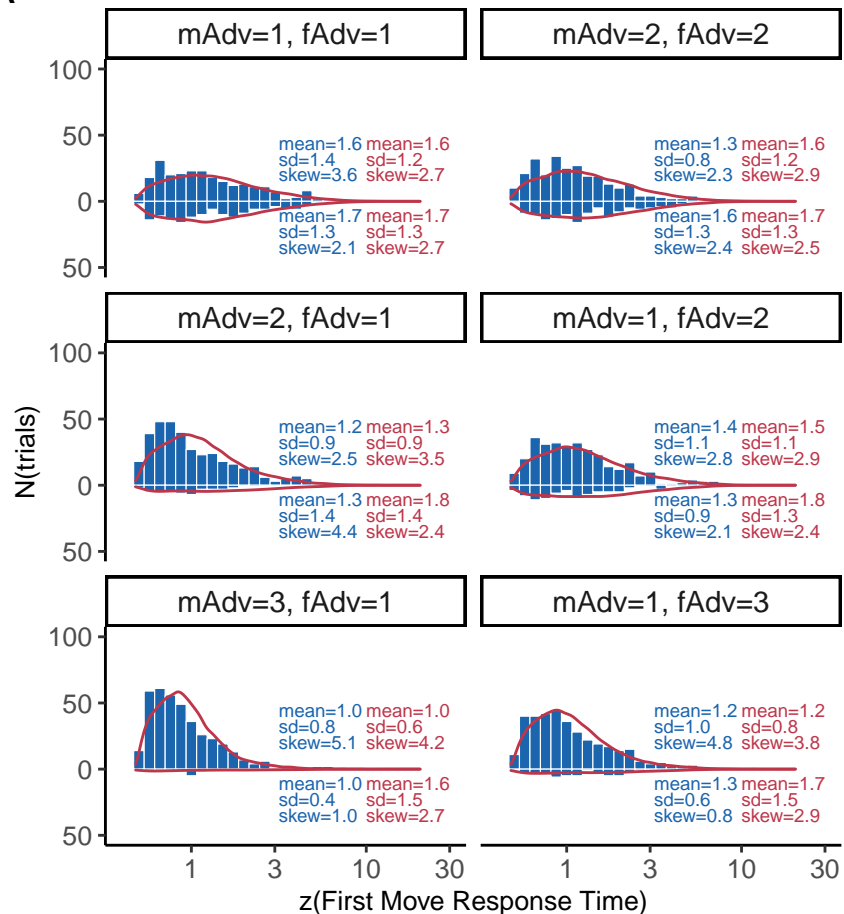

B

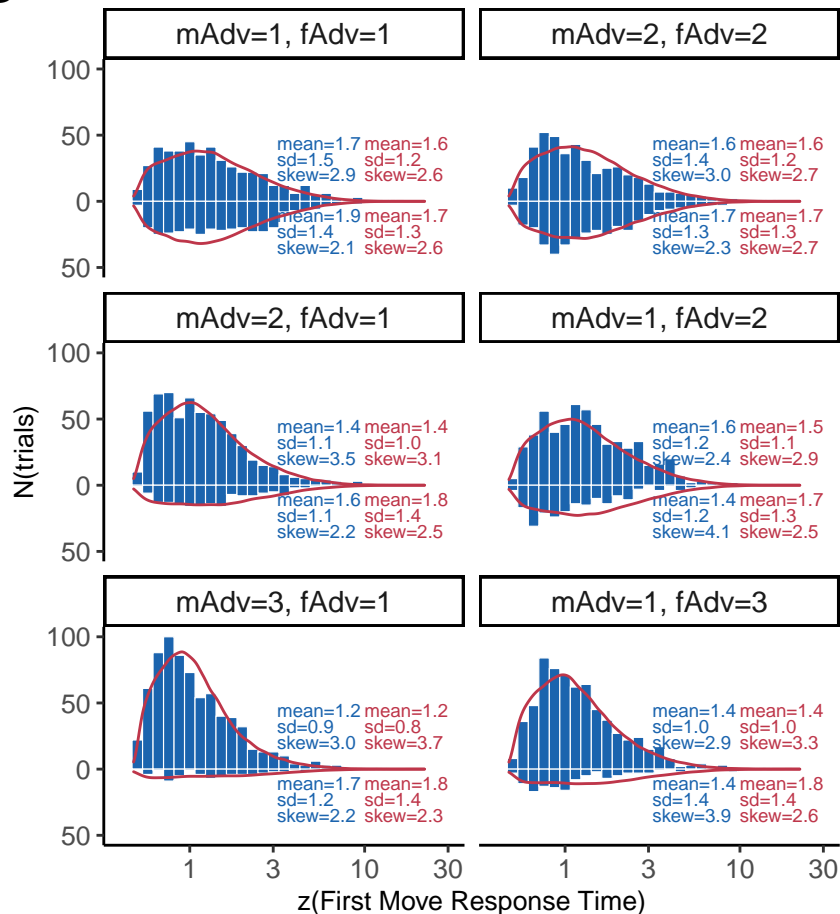

Supplement: S4 Fig — A. Base trials. B. Subgoal trials. Visualization and notations as in Fig 4C. (PDF) [file pcbi.1009553.s005.pdf]

higher accuracy group

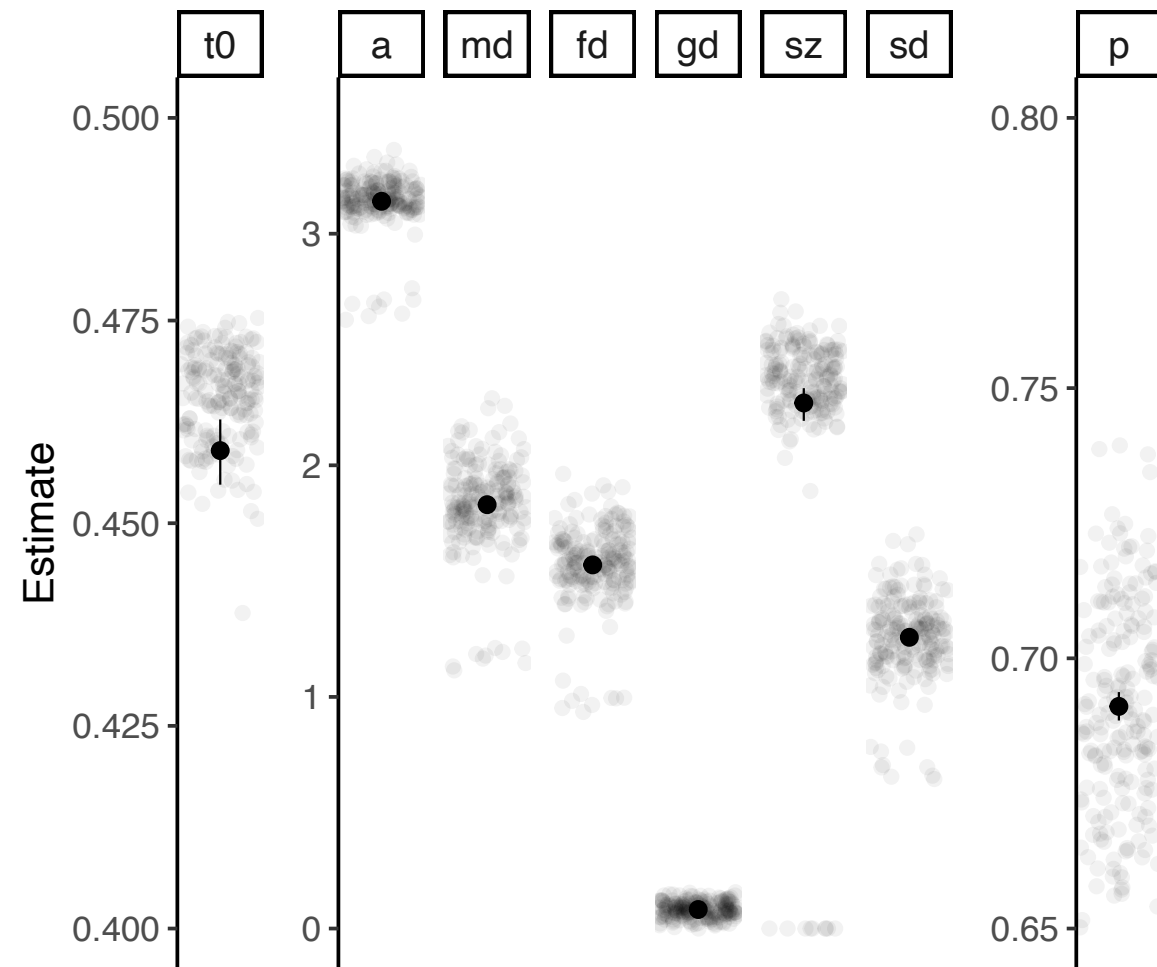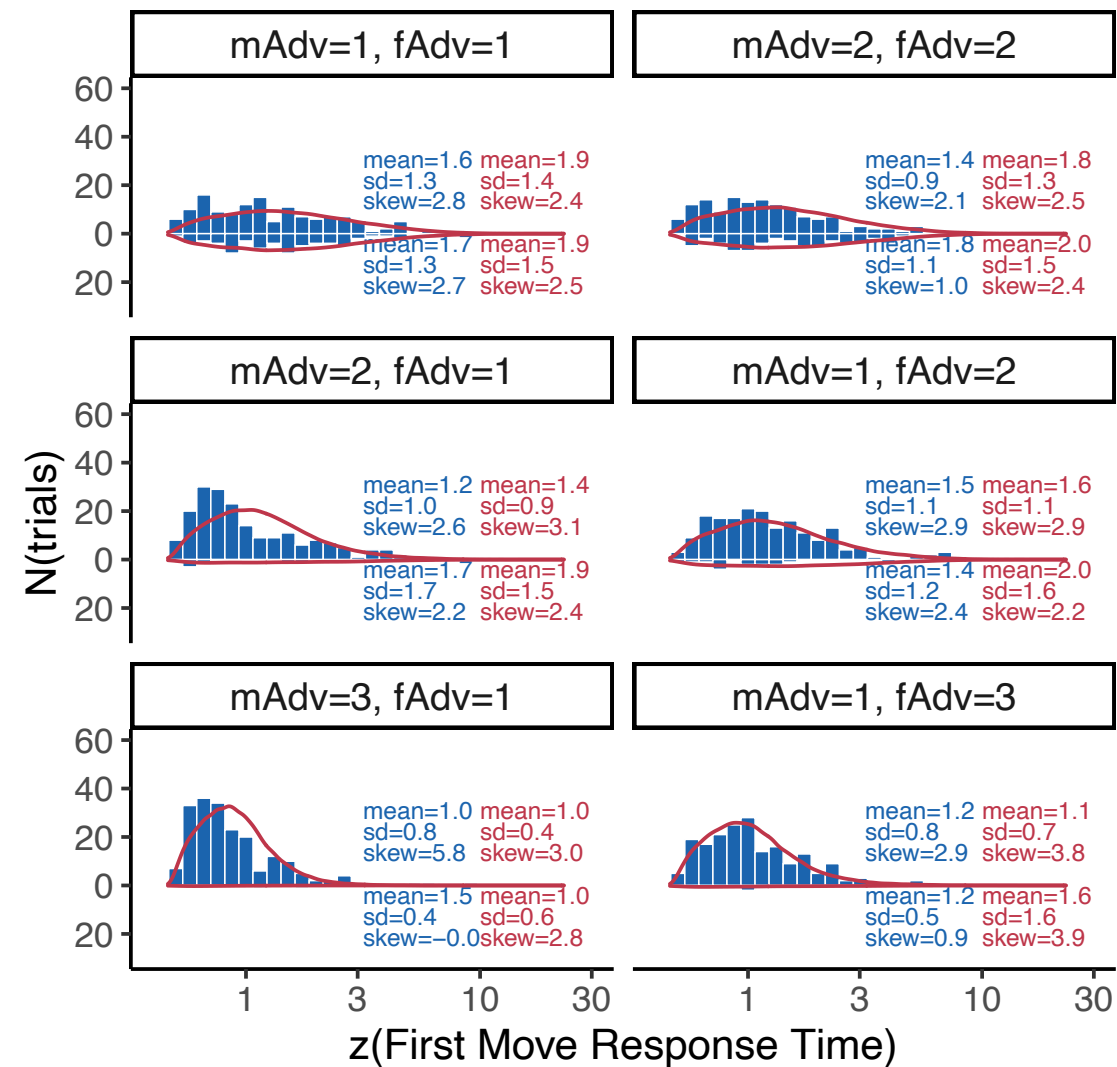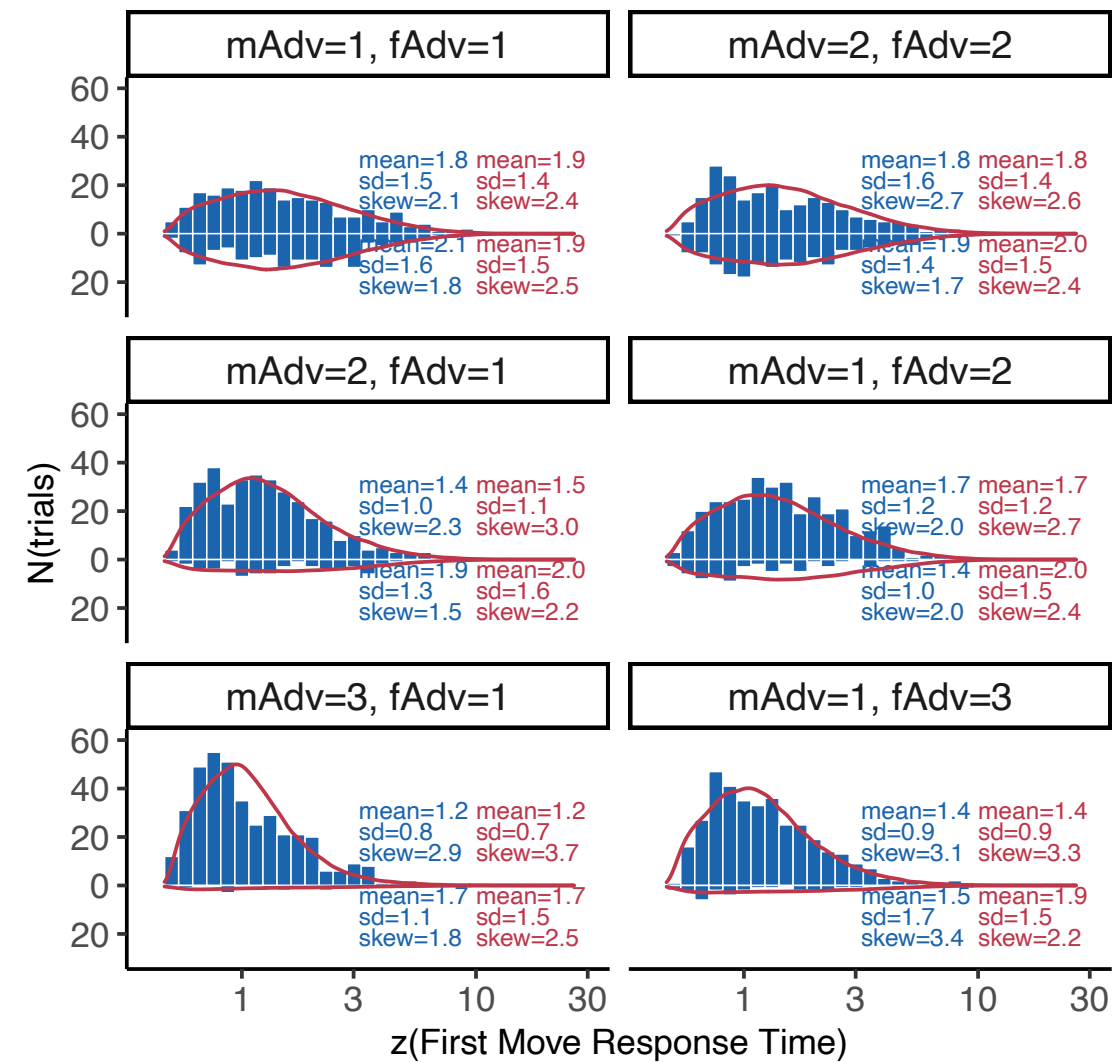

lower accuracy group

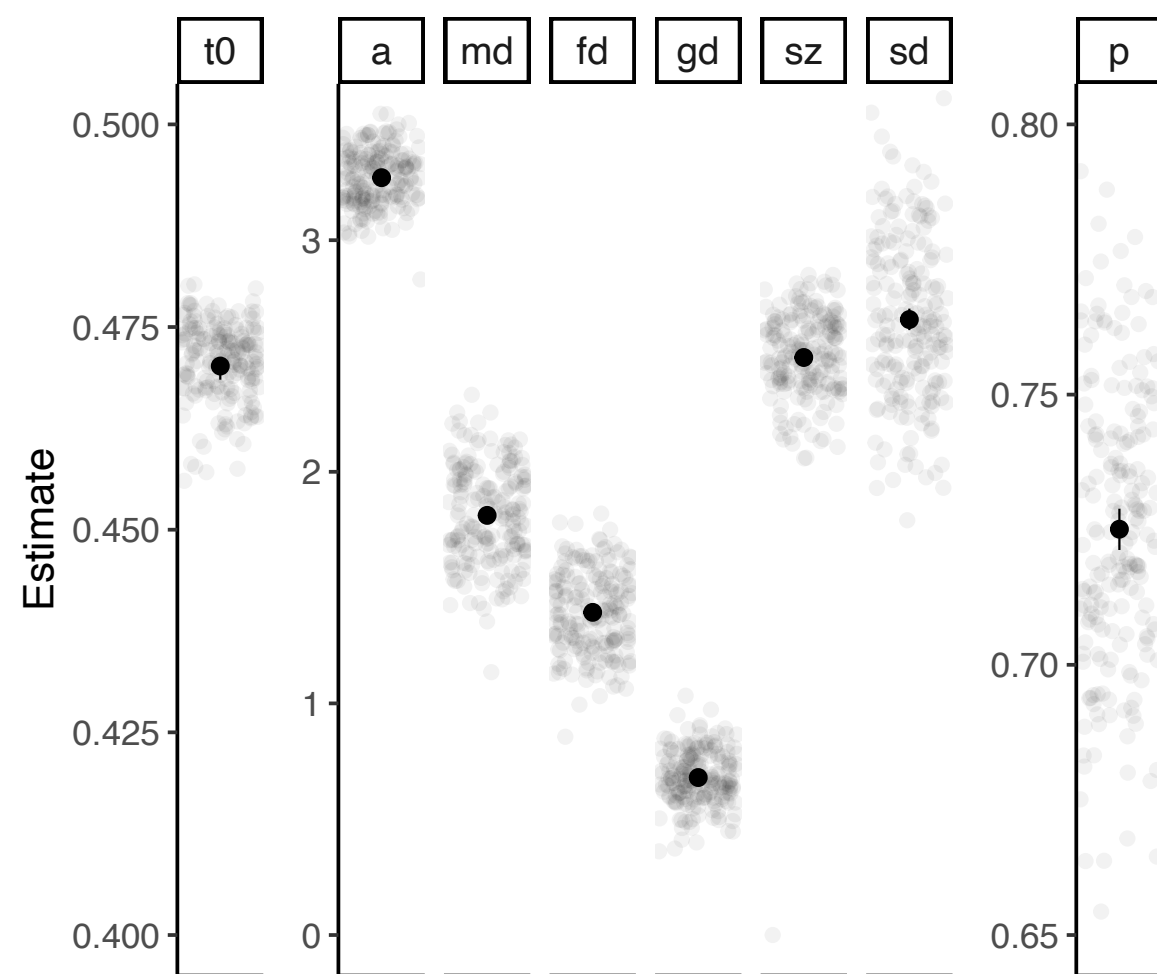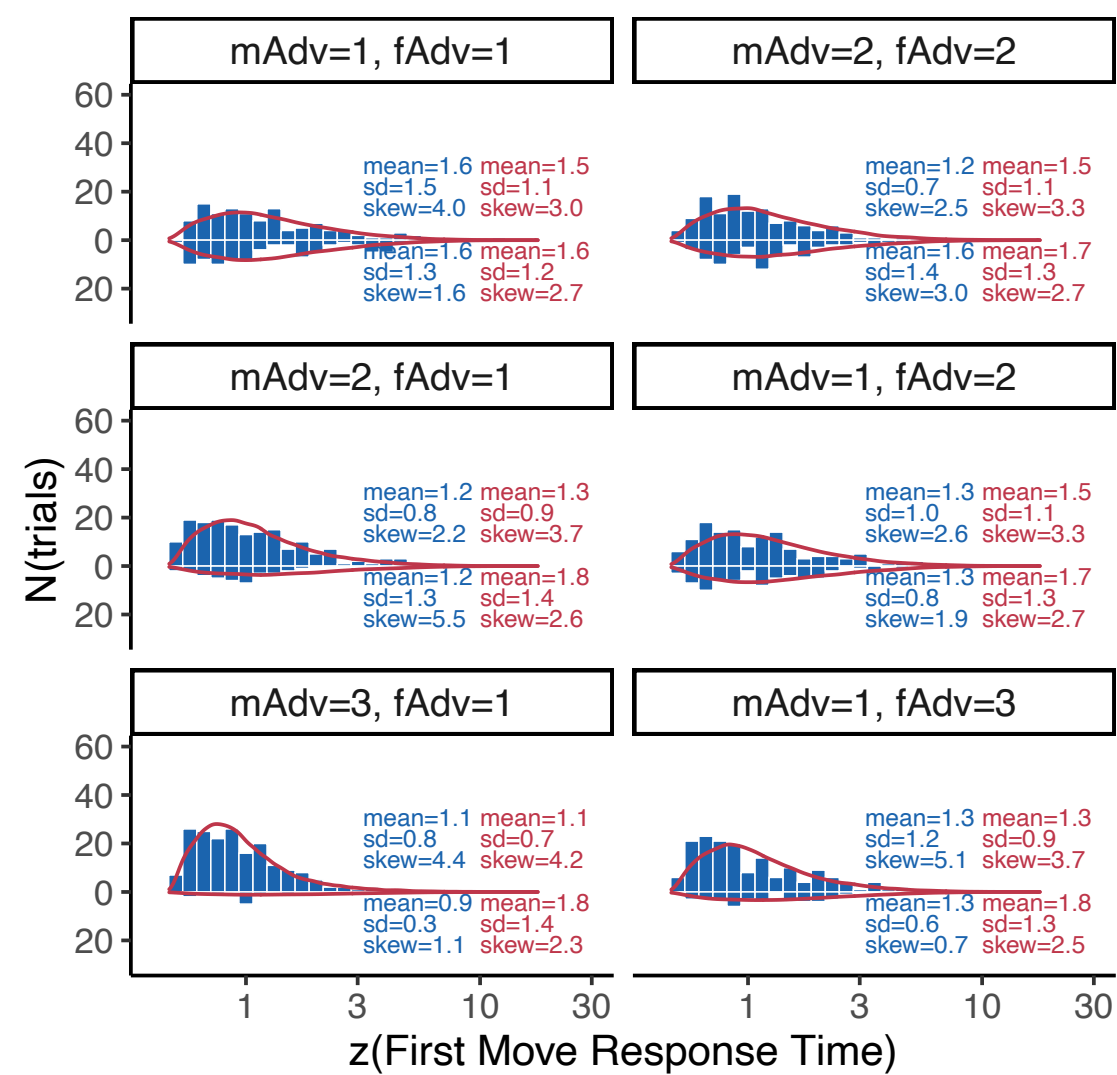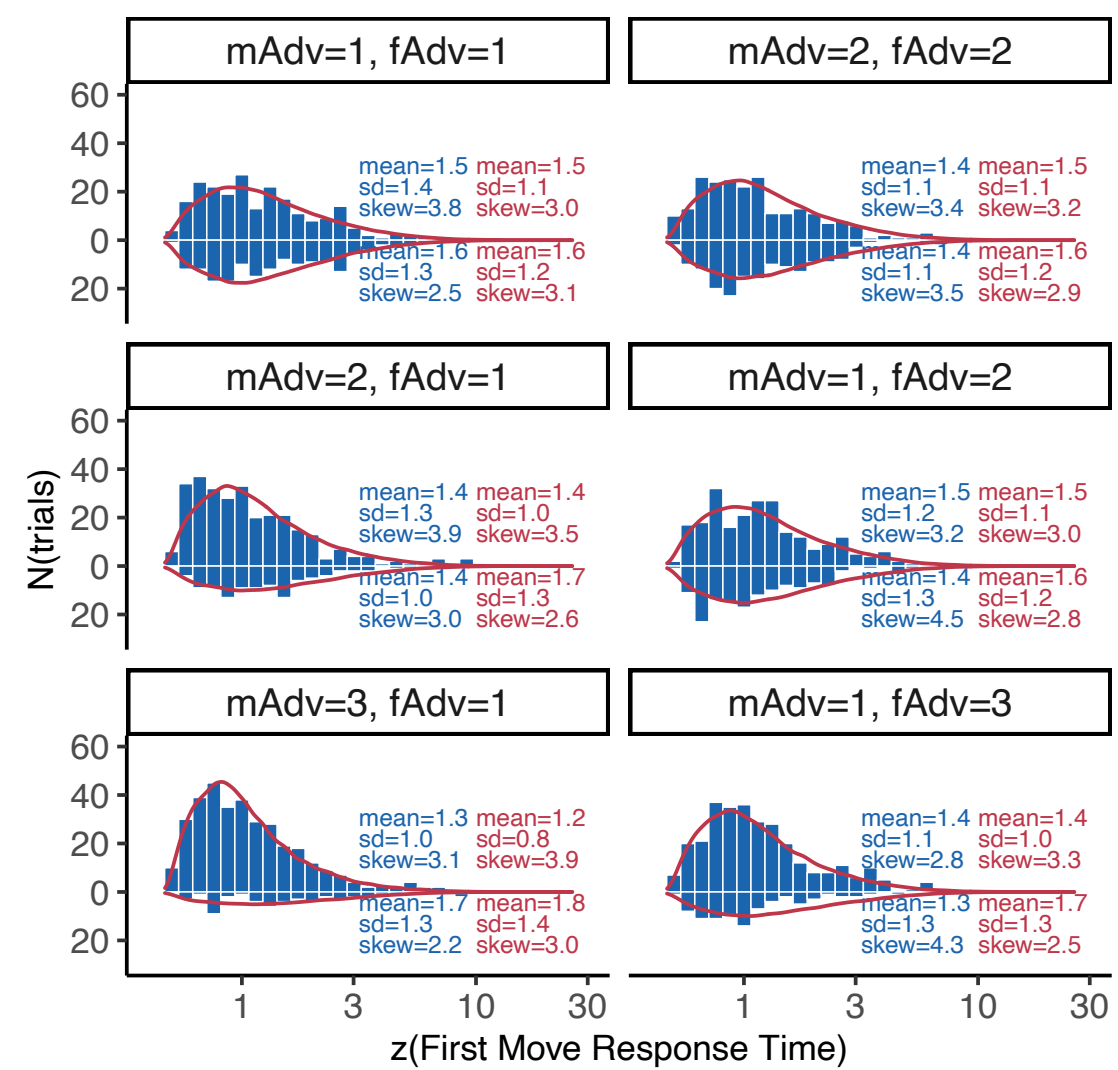

Supplement: S5 Fig — Left panel, model parameter estimates. Middle panel, base trials. Right panel, subgoal trials. Visualization and notations as in Fig 6C and S4 Fig. (PDF) [file pcbi.1009553.s006.pdf]
