# Supplementary material for: A weighted constraint satisfaction approach to human goal-directed decision making
Source: PLoS Comput Biol. 2022 Jun 16;18(6):e1009553. doi: 10.1371/journal.pcbi.1009553 (PMC9255770; doi:10.1371/journal.pcbi.1009553)
Supplement: S6 Fig — Participants’ self-reported considerations of different task elements was in line with the behavioral analyses presented above. In free-form responses, when asked how they came up with the route to reach the goal, their responses consistently suggested that the reasoning process was rapid and intuitive. Likert scale: 1=never, 2=rarely, 3=sometimes, 4=often, 5=always. Sprite denotes the movable blue block. Error bars indicate bootstrapped 95% confidence limits. (PDF) [file pcbi.1009553.s007.pdf]

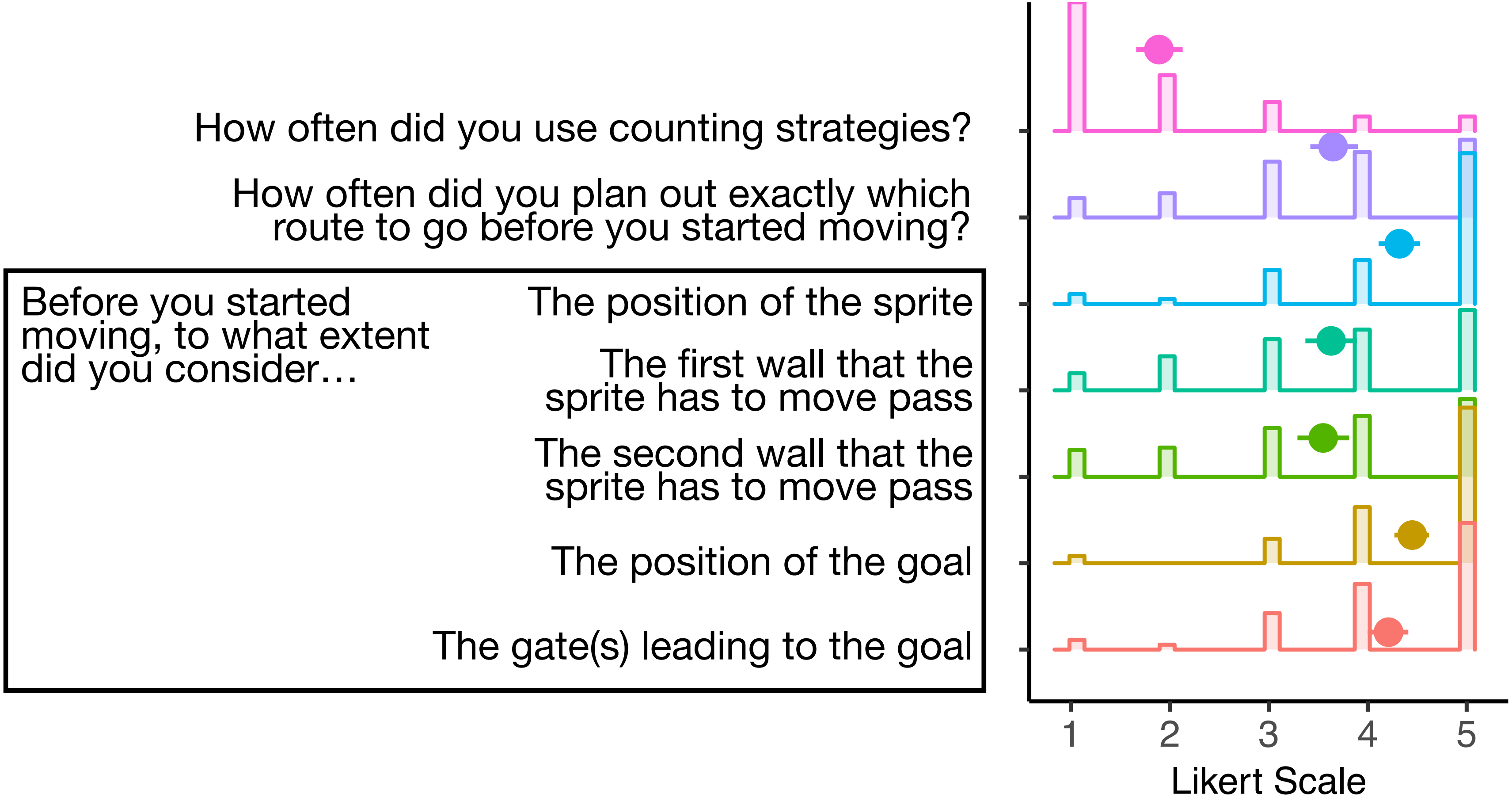

How often did you use counting strategies?
How often did you plan out exactly which route to go before you started moving?
Before you started moving, to what extent did you consider…
The position of the sprite
The first wall that the sprite has to move pass
The second wall that the sprite has to move pass
The position of the goal
The gate(s) leading to the goal
1
2
3
4
5
Likert Scale
