# Supplementary material for: A weighted constraint satisfaction approach to human goal-directed decision making
Source: PLoS Comput Biol. 2022 Jun 16;18(6):e1009553. doi: 10.1371/journal.pcbi.1009553 (PMC9255770; doi:10.1371/journal.pcbi.1009553)
Supplement: Attachment — Submitted filename: response to reviewers.pdf [file pcbi.1009553.s008.pdf]

Reviewer #1: Li & McClelland present two experiments combined with drift-diffusion modeling asking how people weigh distal and proximal constraints while navigating towards a goal. The problem is interesting and timely, but I am afraid the paper suffers from a number of quite severe issues.

First, unfortunately I found most of the exposition quite incomprehensible despite being well versed in both drift-diffusion modeling and reinforcement learning (which although not used here directly, the authors use for motivation). This was due two broad categories of issues: 1) many ideas and terms specific to the project were used matter-of-factly before they were introduced or adequately explained, and 2) some details were missing entirely.

We thank the reviewer for raising these points. In our revised manuscript, we have 1) made sure the key terms were introduced and internally consistent across sections, and 2) added details of the modeling methods to clarify our particular approach. We hope that our revised manuscript addressed these two issues.

For example, it was never entirely clear to me how the weighting is actually implemented, which is a core topic of the paper. From the plots I could infer that it was based on the drift rate, but what kind of function of the drift rate? Is it just a separate single drift rate estimated for each condition? If so, how does the distal goal come into play in Exp 2? The full modeling assumptions are unclear.

We have significantly revised the drift-diffusion modeling section in the Methods in both Experiment 1 and 2 to clarify how we used the drift-diffusion framework to interpret our data. The newly added equation on line 218 clarifies that the mean drift-rate for each trial is an aggregated value based on the weights (free parameters) and the magnitude (experimental-defined value) of the optimality-relevant constraints in our task. In Experiment 2, we modeled separate drift rates for the base task and the more complex subgoal task by comparing model variants where the weights of the optimality-relevant constraints change in different ways, as well as adding a third additive component to the drift rate representing the weight of the distal goal.

Mixed-effects are mentioned, but the details are scattered and hard to piece together.

In the revised manuscript, we have clarified how the mixed-effects choice model is a different analysis from the drift-diffusion model. At the beginning of both Results sections, we included a mixed-effects choice model and a linear response time model to provide statistical support for our findings on the overall choice optimality and response time patterns in the entire dataset. The drift-diffusion models were fitted on a subset of the data, and were intended to test the two hypothesized planning processes (hierarchical task decomposition vs. weighted constraint satisfaction) in a way that is informed simultaneously by the choice and the response time data. Within the drift-diffusion modeling analysis, we implemented another mixed-effects model to compare the performance of different model variants on held-out test data across all cross-validation folds.

One sentence of the methods reads "The walls on the filler trials were of length- 3, 5, or 7 and were sometimes shifted from the center..". When is "sometimes"? What is the algorithm for designing the state space?

We added a more full description of the Experiment 1 filler trials on line 193.

I thought carefully about how to phrase the following, but I think it's fair to say the paper needs to be re-worked essentially from scratch, carefully including all details and slowly building up the questions and their answers.

We thank the reviewer again for sharing these comments. We hope the revised manuscript has clarified these points and presents a more comprehensive build-up to the key issue and motivates our particular approach.

Second, related and more importantly, the specific scientific questions being asked were themselves not altogether clear, and may represent a strawman. For one, I wasn't sure whether a claim was being made about participants using a hierarchical representation for this particular task. The introduction suggests thinking about these problems in terms of hierarchical reinforcement learning, and as far as I could tell, the idea being that people might select an abstract sub-goal but then still be distracted by high-level features of the state space (the ultimate goal) even after they "step down" one level to deal with the subgoal. But, there is no evidence presented that people use a hierarchical representation in this task at all.

After considering these comments carefully, we recognize that some of the statements in the paper were unclear in characterizing our stance on whether participants are actually using some form of hierarchical decomposition. After further discussions between ourselves, we have clarified our own perspective on this matter.

We are not claiming that people use a hierarchical representation either explicitly or in some latent way. Instead, we are exploring alternative accounts of behavior in a setting where the overall optimal path choice depends on taking the myopic and future constraints into account while ignoring the final goal position. We are arguing that behavior approximately consistent with the optimal behavior can be achieved through the stronger weighting of information relevant to the embedded subtask (finding the path to the subgoal) relative to the smaller weighting of information irrelevant to the subtask (the final goal). This is revealed through the biasing influence from the irrelevant final goal in choosing the initial step, as the reviewer noted. This is in contrast to a strictly hierarchical reduction of the overall task into the smaller subtask when choosing the first action, because the lower-level subtask was not treated in an independent context without influences from out-of-context factors. Therefore, we think of this as evidence that people do not deploy encapsulated subtask executions when smaller subtasks are embedded in larger task contexts (like when an option-specific context is activated in the hierarchical reinforcement learning framework), but that they approximate this optimal reduction process through modulating the weighting of different pieces of information in that direction.

If they do [use a hierarchical representation], it is also important to understand *what* representation is being used. There is no reason to think that the only subgoal location would be at the door. For example, participants may create subgoals directly on each side of a barrier. A question then is whether it is the *decomposition* that is interfering with decision making (i.e. maybe they chose a poor decomposition) rather than (or in addition to) at an algorithmic level whether participants reason across or within modules of the decomposition appropriately.

We agree that there are alternative possible construals of the subgoal structure of our base task – as the reviewer notes, it could be construed as having several subgoals. Our main focus is on how performance within the base task is affected when it is embedded in the more complex task as a subgoal. However, we do note the following: Since the base and the complex task are both set up so that optimal initial choices need to be based on the joint influence of the two base-task-relevant constraints (the myopic and the future advantage, in our terminology) it seems most natural here to us to think in terms of a joint, and perhaps simultaneous, consideration of these two constraints.

Putting hierarchy aside, one may interpret the paper as asking about how people weigh proximal vs distal constraints, but from this perspective the results appear to be entirely unsurprising: people are worse at the harder problem in Experiment 2 and have a myopic bias. These results in themselves unfortunately do not add anything new.

We thank the reviewer for giving us an opportunity to think more about how to make clear what we hope to be contributing to our understanding of planning and decision making. We agree that the greater weighting of proximal over distal constraints in and of itself is certainly not a new finding. Such a tendency to discount the future can be regarded as the general context in which we investigate a further question – the idea that modulation of this baseline weighting can characterize participants' decision making in our tasks, and further, that this approach can serve as a useful alternative to a hierarchical task decomposition approach. Within this framework, we argue that people optimize their path selections by modulating the constraint weightings in the direction that enhances optimality. In support of this, we find that more accurate participants weight the myopic and future constraints more strongly and more equally than less accurate participants, and that they place less weight on the irrelevant final goal position than the less accurate participants. We believe our findings will help motivate others to consider alternatives to hierarchical task decomposition-based approaches to planning and decision making, and we point the way toward mechanistic models that would implement the weighting of constraints through the context-sensitive allocation of attention. We hope the revised manuscript (especially the General Discussion section) makes these ideas more clear.

Reviewer #2: The paper “A weighted constraint satisfaction approach to human goal-directed decision making” by authors Li & McClelland investigates how reaction time and accuracy change when participants navigate to a sub-goal in a maze (the “sub-goal” condition) compared to when this sub-goal was not embedded in a higher-level structure and the only goal in the task (the “base” condition). The key comparison relates to the influence of path “advantages” where it was easier either to turn around an obstacle from the start, at the goal, or both. The results using drift diffusion modelling show that while participants assign high weight to these advantages in the base condition, seemingly trading off these two components, their performance is slightly impaired in the subgoal condition, albeit with similar relative weighting between advantages. The paper concludes that this provides evidence for a parallel consideration of planning constraints.

The paper is well written and presents a clever paradigm in which drift diffusion modelling can illuminate planning behaviour. While I would be happy for this paper to be published in its current form, I do have some concerns relating to interpretation and terminology and would appreciate it if these were addressed or clarified in a revised manuscript. But do pick and choose from these as you please.

Major:

1. I’m not sure the results directly map onto the sequential/parallel distinction made in Fig. 1. It is unclear to me how the finding that the goal in experiment 2 influences myopic advantage relates to parallel planning. Wouldn’t a claim that this shows “forward” planning rather than “backward” (from the goal) be equally supported without requiring this additional claim?

Our particular modeling approach does not directly speak to the directionality of the planning process. We thus focus more on how much each factor is weighted in decision making when they are all considered to some extent in deciding the first step, and we do not make further inferences about what explicit strategies may have given rise to these weights. We hope the presentation in the current version is clearer in highlighting the central distinction we are making between a hierarchical task decomposition vs a weighted constraint satisfaction approach. Please also refer to our response to Reviewer 3’s point 4 for related comments.

2. I also wonder in how far this “myopic” bias in the sub-goal condition is favoured by the goal being clearly visible, whereas planning to the sub-goal (and working out its advantage) seems more difficult when the sub-goal is only indirectly marked using the bottleneck next to it. My hunch would be that if the sub-goal were marked, planning would look less affected by the irrelevant goal position. In other words, is this mainly an effect of difficulty because the sub-goal needs to be inferred? This is particularly important as trials were interleaved as far as I understand it. Are there perhaps enough consecutive sub-goal trials to check if participants improved within such a mini-sequence?

The reviewer raises an interesting point about the possible difference in salience between the subgoal location in the complex task condition vs. the goal location in the base task. However,

we did not find an increased weighting of the myopic relative to the future advantage in the complex task – the influences of both constraints were degraded by about the same amount in the complex task compared to the base task, suggesting that the use of all subgoal-relevant constraints is degraded by the overall complexity of the more complex task and/or the importance of reaching the final goal.

We do agree with the general point that the more complex display that participants must process in the more complex task setting may be a factor contributing to the proportional degradation of both the myopic and the future constraint weights. In this connection it is noteworthy that the myopic and future constraint weights were degraded to an approximately equal extent for both the more accurate and less accurate participants, suggesting that this degradation is not as subject to modulation as the relative weighting of myopic vs future or the degree of influence of the irrelevant final goal.

Minor:

3. I have some quibbles about the “advantage” and “constraint-satisfaction” terminology. I see that advantage favours either forward or backward planning in the base condition, but I found this term unintuitive at first, because for the purpose of the task there is no advantage at all, just a biasing influence to take an easy way around the obstacle (and thus possibly make it easier to find the optimal route). Perhaps a term like “salience” could be more appropriate?
4. Similarly, my understanding is that constraints often mean something a bit different in the literature, e.g. budget constraints in economics, or time-constraints in navigation, for example in a similar setting in (Ringstrom and Schrater, 2019). In the present study, the constraints are essentially the maze layout (boundaries and bottlenecks), which are seldom referred to as constraints in other studies. Nevertheless, I agree that the distinction between having to overcome the barriers near the start and the goal is interesting and relevant. However, I would see this more in terms of “chunking”, or “options” within the Reinforcement Learning framework (Precup et al., 1998) rather than constraints. And in my personal view only the multi-subgoal task fits the more conventional sense of a constraint. What was the reasoning for not including this trial-type in the analyses?

Combined response to points 3 and 4:

We have clarified our use of the words “constraint” and “advantage” in the revised manuscript. Viewed locally, the quantitative myopic and the future path advantages often do signal an advantage of one path over another (with regard to the objective of finding the shortest goal-reaching path), and when viewed in the entire task context they need to be considered together before committing to a first-step choice. The two advantage types (myopic vs. future) only lead to no relative advantage for one initial path direction over the other in a small subset of the trials (i.e., when both paths are equally optimal).

We use the word “constraint” to specifically refer to the influencing role of the abstracted-out factors in the task (i.e., myopic and future advantages, the final goal) on participants’ decision

outcome and decision processes. This is different from the idea of “chunking” and “options”, which are terms that refer to construals of the partitioning of the problem space. In other words, we use constraint to refer to factors that can potentially contribute to participants’ decision making, whether or not the factor can be viewed as residing in a particular chunked subspace of the problem, or within an activated option context. The notion of constraint satisfaction is also consistent with other cognitive processes considered in the PDP literature in which we motivate our account in the context of goal-directed decision making.

Responding to the reviewer’s question at the end of point 4, please refer to our response to Reviewer 3 about the multi-subgoal trials.

5. I found Figs 3 and 5 to be quite busy and had trouble working out what the relevant effects were without looking at the text in detail. Perhaps adding a key statistical test with significance to the plots would help. E.g. the IA-CA comparison referred to in line 223.

We agree the figures contain a lot of information, but are concerned that marking which effects are significant might make them even more cluttered. We have substantially rewritten the text describing the comparisons of interest. We hope that the text now draws attention properly to the relevant comparisons to look for in the figure.

6. I find the claim (lines 441f) that the model does not need to separate problem decomposition and execution a little unconvincing. In the current version the constraints are hard-coded and so the model really only speaks to execution over a pre-decomposed space. Granted I can’t think of a better decomposition for this current task, but any modification of the boundaries would always entail a necessary revision of the components of the model.

The reviewer is right that the critical base and subgoal trials might (at least after a few instances over the first part of the experiment) be recognizable without requiring a separable decomposition step that could be necessary in other settings or on the first trial encountering a novel maze configuration. This is an instance of the general concern that features of the design of the experiments may have influenced the results. We acknowledge this possibility in the discussion, in the paragraph spanning lines 641-653.

As for the model, the reviewer is right that the current model does not serve as an immediately generalizable formulation to explain behavior in other maze configurations. Here, we have abstracted out three task factors (the myopic and future advantages, the final goal) that influenced participants’ choice behavior, and modeling their weighting in decision making has enabled us to show that our weighted constraint satisfaction account can capture behavior in this particular task setting and serve as an alternative to the hierarchical task decomposition approach. Because the two subgoal-relevant constraints need to be jointly considered in initial step choices but the final goal is irrelevant, and because participants overall choose the optimal path on a large percentage of the trials, it turned out that this level of characterization aligns well with the pattern of data. We hope the revised presentation clarifies the rationale for all of the analyses presented, and we also note that we have now fully integrated the fitting of the

influence of the final goal position into our drift diffusion modeling of the results from Experiment 2, as requested by Reviewer 3 and as discussed further below.

References:

Precup, D., Sutton, R., and Singh, S. (1998). Theoretical results on reinforcement learning with temporally abstract options. Machine Learning: ECML-98. Springer Berlin Heidelberg 382–393.

Ringstrom, T.J., and Schrater, P.R. (2019). Constraint Satisfaction Propagation: Non-stationary Policy Synthesis for Temporal Logic Planning. ArXiv.

Reviewer #3: Summary:
This paper reports two experiments and modeling that test the hypothesis that hierarchical planning involves parallel processing of information about longer-term goals even when people are pursuing a sub goal. This claim is contrasted with the idea that processing of sub goals and goals occur sequentially and separately. Exp 1 examines RTs when people navigate simple mazes where they must navigate around two barriers to reach a goal. Start and goal states were placed such that rounding the first barrier from the start or rounding the second barrier to reach the goal were longer or shorter on different trials. By examining reaction times on different trials, this design allows for assessing differential biases to plan based on the configuration around the start versus goal locations. Results for Exp 1 show that people use both start and goal configuration information to plan (as evidenced by the general tendency to take the shortest path that integrates information on both the start and goal sides), but that people have a small bias towards the start configuration. Exp 2 examines RTs when Exp 1 mazes are embedded in larger mazes, such that the original maze is a subtask for completing the new maze. Using differentially parameterized drift diffusion models, they find that the presence of a final goal that should not affect optimal behavior or processing on the subtask influences first-move RTs. Since reaching the final goal is a supertask and reaching the other side of the first room is a subtask, they conclude that this influence indicates that goal processing occurs in parallel with planning to the subgoals.

Main Review:
I enjoyed reading this paper and appreciate the experimental approach taken. The design of the base mazes versus embedded mazes was especially clever and intuitive. However, I have several major concerns about confounds in the experimental design, the space of models tested, and the overall framing of the paper.

1. There seem to be confounds in Exp 2 that need to be controlled for. Importantly, the base and subgoal mazes are not only different tasks, they also differ perceptually (e.g., their complexity and the objects present). The authors' main claim regards how planning in a subtask context is influenced by a supertask context and *not* by just the perceptual differences, but these are both present. Another way to think about it is this: the presence of the red goal block on the subgoal trials (Fig 2) could have influenced subtask processing because it is being maintained as *as a goal in a hierarchy*, or simply because it *perceptually distracting*. From what I understand, the results of Exp. 2 can only support their main argument if the former and not the latter is true, and so this needs to be ruled out. I could not tell if the multiple subgoal trials were introduced to account for this, and the authors note that these data were not analyzed (line 305). Would some analysis of these trials be able to account for the perceptual effect of the red goal? If not, then ideally, the authors could run controls where the base/subgoal trials are perceptually equivalent. One possibility is that participants could be shown the same mazes but told before viewing the maze that they need to get to the star (subgoal version) or just the door (base version). Other variations are also possible.

We thank the reviewer for raising this possible confound. It is true that the visual presence of the final goal may contribute to the effect of the final goal position. However, any visual salience

effect is likely, in our view, to be a joint function of the presence of the goal cue in the display on the test trial and of its relevance within the experiment as a whole – e.g. within the same trials, where it must be attended to as the final target or in different trials as in the reviewer's proposed experiment. Our finding that more accurate participants' path choices on the subgoal trials are less affected by the final goal does not rule out a perceptual salience effect, but does suggest that any such effect is subject to cognitive control, just as the Stroop effect and other task-dependent behaviors appear to be.

2. Another issue involves the space of models tested---in particular, the assumption of hierarchical planning occurring in the task. For Exp 2., the authors modeled their data with drift diffusion models where the drift rate included parameters associated with the start location (the myopic advantage weight), the subgoal location (the future advantage weight), and the goal location (the final goal weight). It sounds like they only fit the goal weight after fitting and then freezing the myopic and future weights (line 396), which roughly corresponds to fixing the parameters of the subtask context and then testing if the supertask context explains additional variance. However, these analyses all presuppose hierarchy---what if people are not planning hierarchically at all and are simply engaged in flat planning? In the DDM modeling framework they use, this corresponds to fitting all three parameters simultaneously (I think). The authors should compare the fit of this model to the fit of the ones already tested (e.g., by reporting delta AIC).

Our original rationale for using a sequential, two-part fitting procedure for Experiment 2 was that the goal weight explains a different dimension of variance in choice and reaction time patterns – we did not intend this to suggest anything about participants' cognitive processes. However, we do agree that considering all of the constraints in a single joint fit would be the best approach, and we thank the reviewer for this suggestion. Accordingly, we have replicated the original results with a synchronous model fitting procedure, where the drift weights for all three factors (the myopic and future advantage and the final goal) were estimated together. The winning model remained the same model, and the test data objective across matching cross-validation folds improved under the synchronous fitting scheme, with Δ sNLL = -1160.575 (SD = 43.75) compared to the original winning model fits. We have updated the results in the revised manuscript accordingly.

3. Additionally, while I appreciate the authors' attempts to determine whether subtask processing is isolated from a full hierarchical context, couldn't interference from the goal just mean people are doing flat planning? Put another way, when people are flat planning, they are planning in a context that includes the entire task (all the constraints, the start, bottleneck, and goal). Other algorithmic considerations could come into play within a single flat planning context---as a concrete example, people could be performing heuristic search using a start-to-goal distance heuristic that biases their initial action or reaction times. I understand that a general challenge with modeling these kinds of tasks is that the space of possible planning algorithms is large. Nonetheless, the authors want to make a fairly strong claim about a class of planning algorithms (ones that involve hierarchical decomposition) but they need to provide evidence that this type of algorithm is being used by participants to make such a claim.

As the reviewer noted, our results suggest that planning is sensitive to the entire context, and indeed they are consistent with a 'flat planning' model, and this is, indeed, what we are suggesting that participants may actually be doing. As stated in the cover letter, we hope the revision is now clearer on this point. Our main proposal here is that task-sensitive modulation of the (flat) weighting of all factors, for example, weighting the subgoal-relevant information more than information outside of the subgoal context, can approximate the optimal behavior generated by a strict hierarchical task decomposition approach. This approach allowed us to capture choice behavior with varying levels of optimality (with the analyses of the individual differences comparing more accurate and less accurate participants). Please also refer to our response to Reviewer 1's second point at the bottom of page 2 in this document.

It is entirely possible that the weighting of the final goal in deciding the first step corresponds to a heuristic strategy of minimizing the distance from the starting point to the goal. Since our focus is on how different factors are weighted in decision making, we do not make inferences about what the explicit strategies may have given rise to these weights beyond the relative magnitude of these different considerations.

4. I found the contrast between serial and parallel processing during hierarchical planning to pose somewhat of a false dichotomy. Even if people are hierarchically decomposing a task, couldn't interference from the goal context be because of switching between goal/subgoal processing (i.e., multiplexing)?

Our main focus on the sequential aspect of the hierarchical planning approach is whether an independent initial stage of task reduction necessarily precedes action selection. The winning drift-diffusion model is consistent with the view that a relatively stronger weighting of subgoal-relevant information compared to the subgoal-irrelevant final goal enabled an approximation to the optimal behavior predicted by a hierarchical task decomposition approach.

It is possible that the overall parallel processing of multiple factors is an aggregated effect from more discrete processing of different factors (as the reviewer suggests, multiplexing). However, with choice and reaction time as the available behavioral observation, our particular approach of modeling the decision as a drift-diffusion process does not grant us the ability to robustly infer that kind of characterization of the underlying decision process. We now explicitly acknowledge this possibility in the discussion section (lines 587-605), suggesting that future research monitoring eye movements could potentially shed light on this issue, and citing studies that have done this to useful effect.

Minor points

- At several points, the authors claim that parallel processing is "the optimal approach" (e.g., line 68). It is not clear what this means---optimal with respect to what?

We meant that with respect to the objective of choosing the shortest goal-reaching path, the optimal approach to accurately selecting the first action (and thus the initial path direction)

needs to rely on the joint consideration of the myopic and the future constraints before acting. We have clarified this in the revised introduction when we discuss the base task (line 61).

- Figure 3 - why use probit transform? couldn't you just show the probabilities?

We opted for the probit transform as it allowed better separation of the data points (since in probability space they are close to the ceiling), as well as for better alignment of our use of the probit linking function in the generalized linear mixed-effects model for the choice data across all trials.

- Lines 315-317 - the multi goal trials seem critical but I'm not sure what these sentences are saying.

We have clarified the description of these trials in the revision. We included them in running the experiment to address a subsidiary question that is not the focus of the current paper. More details of these trials can be found in our OSF repository. For completeness, we show an example of these trials below. We have yet to conduct a full analysis of these data. We can, however, report that initial path choices differ reliably between the single-subgoal trails like the one illustrated on the left vs the multi-subgoal trials illustrated in the remaining panels. In the leftmost panel, the optimal path favors the future advantage based on the position of the single entry point to the chamber containing the final goal, and participants tend to choose this path about 75% of the time. In the other panels, where there are two entry points to the chamber containing the final goal and both the starting-upward and starting-downward paths are equal in length, participants' responses are nearly equally balanced between the two path directions.

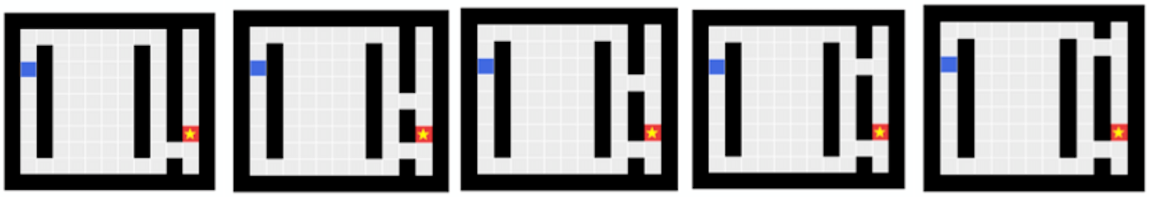

- It seems like a DDM model was used in this work because it can account for reaction times, however, work on planning and hierarchical planning is generally done with markov decision processes (MDPs; e.g., the optimal behavioral hierarchy work by Solway et al., 2015, other work by Botvinick and colleagues). Some discussion of why DDMs were used and not MDPs would be helpful here, especially since the DDMs are not used to model planning per se but to more abstractly model an integration process.
- From what I can tell, choice data and reaction times were analyzed separately (the former with hierarchical probit models, the latter with DDM models). Would it be worthwhile to analyze them together in a DDM model? This type of analysis might be able to reveal speed/optimality tradeoffs.

Combined response to the above points:

The probit choice optimality model (and the linear response time model) discussed at the beginning of both Exp1 and Exp2 results section were meant to provide statistical support of the characterization of the data in all trials, allowing us to highlight the influencing factors in participants decision making. Regarding the DDM modeling, the reviewer's suggestion is in fact what we actually did, and we hope we have made this clear in the revision: We use DDM to jointly model choice and reaction time on the IA trials (predicting trial-level choices is a part of the objective of the DDM fitting process).

By leveraging the DDM framework, we can effectively use both the choice and reaction time data to constrain our comparison of the two contrasting planning processes (weighted constraint satisfaction vs. hierarchical task decomposition) in terms of different changes in stages of non-decision time and decision computation. We believe that our approach provides a complementary view of human planning to existing approaches such as the MDP framework. Our work shows that planning and decision making across levels of optimality can be captured as an information integration process, putting planning and decision making on a common ground with other cognitive domains such as perception and memory. We have highlighted these thoughts in the revised manuscript as well. Please also refer to our response to Reviewer 2's point 4 for related comments.
