# Supplementary material for: A weighted constraint satisfaction approach to human goal-directed decision making
Source: PLoS Comput Biol. 2022 Jun 16;18(6):e1009553. doi: 10.1371/journal.pcbi.1009553 (PMC9255770; doi:10.1371/journal.pcbi.1009553)
Supplement: Attachment — Submitted filename: response to reviewers.pdf [file pcbi.1009553.s009.pdf]

Reviewer #1: The revision is significantly improved and has addressed my concerns.

Reviewer #2: I thank the authors for fully engaging with my concerns as well as those of the other reviewers. The manuscript has been substantially rewritten and is much improved as a result. I have no outstanding or additional major comments and am happy to recommend the revised version for publication.

Reviewer #3: I appreciate the changes the authors have made to clarify aspects of the paper that were confusing. Below are my responses to the numbered points I made originally:

1. The authors agreed with the possible perceptual confound I pointed out, but they argue that this is unlikely to entirely explain their pattern of results. In support of this interpretation, they write, "Our finding that more accurate participants' path choices on the subgoal trials are less affected by the final goal does not rule out a perceptual salience effect, but does suggest that any such effect is subject to cognitive control, just as the Stroop effect and other task-dependent behaviors appear to be." I'm having a hard time following the logic of this sentence. For example, my understanding was that the effect of the final goal is being operationalized by measuring path choice accuracy/optimality, but they are indicating that this is a finding in itself (unless this refers to an analysis of RTs?). Is the idea that the pattern of responses for a subset of participants (the more accurate ones) indicates that attention to the goal is being modulated? If so, this needs to be spelled out more clearly in a response as well as in the paper itself.

I also want to re-emphasize my belief that although not strictly necessary, running control studies would be the cleanest and most direct way to rule out the perceptual confound. Such studies, if they show a weaker influence of a goal-like stimulus than an actual goal, would greatly strengthen and substantiate their central claim.

Response: We agree that further research in which a final, locally-irrelevant goal that is only held in mind and not visible on a screen would be worthwhile to pursue, and we are grateful that the reviewer does not see this as strictly necessary. We also appreciate the opportunity to further clarify our perspective on these matters. These comments prompted us to express more fully our view of the relationship between weightings of various factors, cognitive control, and the behavioral outcomes observed in our experiments.

To speak more specifically to the reviewer's points: Our individual difference analyses were indeed both a choice analysis and an RT analysis as they were based on the drift-diffusion parameter estimates. Because the weight of each factor influences the decision drifting process, it has an effect on both the resulting RT and the choices, and its value after fitting is thus constrained by both the choice patterns and the RT distribution associated with the two accuracy subgroups. This is also a feature of the role of cognitive control in modulating task performance in the Cohen, Dunbar & McClelland (1990) model of the Stroop effect, where the modulation of control affects both speed and accuracy.

The reviewer mentioned “Is the idea that the pattern of responses for a subset of participants (the more accurate ones) indicates that attention to the goal is being modulated?”, this is one way of putting what we were trying to say, if “attention to the goal” is defined as the amount of weight the goal exerts on the decision process. The estimated weight of the final goal in the subgoal trials would represent the weight of the influence of the final goal, as determined by the full range of associated factors, including the fact that it designates the final goal, its perceptual salience, the fact that it is relevant to initial path choices on the base trials, and other aspects. That more accurate participants exhibited less influence from the final goal (as captured by the smaller value of the goal weight in the model) thus indicates that the influence of the final goal is subject to modulation in service of generating optimal choices. We have incorporated these points in the General Discussion (lines 660-677) and added a new section to relate the findings to cognitive control and task modulation of the weighting of different factors (lines 638-658).

2. I appreciate the authors running this new analysis.

3. I have read the response to R1 that the authors mention and think it sounds sensible. It does however raise the question of what role the hierarchical decomposition theory is playing in this paper as it seems to be a straw model.

Response: Hierarchical models are ubiquitous in research on planning and sequential action, and we feel it is helpful to show that the predictions of a simple hierarchical model are violated under the conditions of our experiment. We acknowledge that hierarchical task decomposition occurs in some situations (line 46-49) and we consider the role of the hierarchical task decomposition approach in the General Discussion. Our overall stance is outlined in lines 591-594: “We do not intend to suggest that explicit task decomposition never occurs. However, even when it does, it may still be the case that information not strictly necessary within a subtask context contributes a graded influence within the subtask context.”. We also mention how our account can be potentially integrated with other popular instantiations of the hierarchical task decomposition account, including reinforcement learning and Bayesian modeling approaches (lines 724-729).

4. I appreciate that the authors have clarified this point.
